# Prosthesis-patient mismatch after mitral valve replacement: a single-centered retrospective analysis in East China

**DOI:** 10.1186/s13019-018-0788-4

**Published:** 2018-10-03

**Authors:** Armah M Akuffu, Haige Zhao, Junnan Zheng, Yiming Ni

**Affiliations:** Department of Cardiothoracic Surgery, the First Affiliated Hospital of Zhejiang University, No.79 Qingchun Road, Hangzhou, 310003 China

**Keywords:** Prosthesis-patient mismatch, Mitral valve replacement, Effective orifice area, Short-term mortality

## Abstract

**Background:**

Prosthesis–patient mismatch (PPM) may affect the clinical outcomes of patients undergoing mitral valve replacement (MVR) surgery. We aimed to investigate the incidence of PPM of the mitral position in our center and analyze the possible predictors of PPM as well as its effect on short-term outcomes.

**Methods:**

We retrospectively examined all consecutive patients with isolated or concomitant MVR at our center from 2013 to 2015. PPM was defined as an indexed effective orifice area (iEOA) of ≤1.2 cm2/m2. After inclusion and exclusion, a total of 1067 patients were analyzed. The baseline information were collected and compared between the two groups. Multivariate logistic regression analysis was conducted to determine the preoperative predictors of PPM as well as the effect of PPM on early mortality.

**Results:**

A total of 1067 patients were included in the study. PPM was detected in 15.9% of the patients while 12 patients (1.12%) met the criteria for severe PPM. Patients with PPM compared to the non-PPM patients had higher age, larger body surface area and were more likely to be male and obese. Logistic regression analysis showed that higher age, larger BSA, bioprosthesis and smaller left ventricle end-diastolic diameter were predictors of PPM. There were no significant differences between the PPM and non-PPM groups regarding post-operative complications. Logistic regression analysis showed that PPM was not a risk factor of short-term mortality (*P* = 0.654). Also, there were no significant differences regarding short−/mid-term heart function between the PPM and non PPM groups (*P* = 0.902).

**Conclusions:**

Our results demonstrated that higher age, bioprosthesis, larger BSA and smaller left ventricle size were associated with mitral PPM. However, PPM was not associated with poorer early outcomes after MVR surgery. In eastern of China, the prevalence of mitral valve stenosis is high; therefore, whether the standard PPM criteria are suitable for patients of this district needs to be further verified.

## Background

The phenomenon of prosthesis-patient mismatch (PPM) was initially described by Rahimtoola and Murphy about 40 years ago [1, 2]. Currently, PPM is considered a condition in which the effective orifice area (EOA) of the implanted valve prosthesis does not match the patient’s body size.

PPM of the aortic position has been proved to be associated with poorer outcomes including long- and short-term cardiac death [3–5]. However, PPM following mitral valve replacement (MVR) has still been less investigated.

In recent years, PPM after MVR has attracted more and more attention from researchers. Researches has shown that the EOA of mitral valve prosthesis is often too small in relation to body size, thus, normally functioning mitral prosthesis often has relatively high transvalvular gradients similar to those found in mild to moderate mitral valve stenosis patients [6–10].

In East Asia, where rheumatic mitral valve stenosis is very common, the mitral valve annulus in patients is relatively small; therefore more patients meet the standard of mitral PPM [11]. We aim to investigate the incidence of PPM of the mitral position in our center and analyze the possible predictors of PPM as well as its effect on short-term outcomes. We will also discuss the eligibility of the current PPM standard for this population.

## Methods

### Patient population and data collection

We retrospectively reviewed all consecutive patients who underwent elective isolated or concomitant MVR at our center, the Department of Cardiothoracic Surgery, the First Affiliated Hospital, Zhejiang University, School of Medicine, from January 2013 to December 2015. Written informed consent waivers obtained from the Hospital Review Board were completed by all patients.

**Fig. 1:**
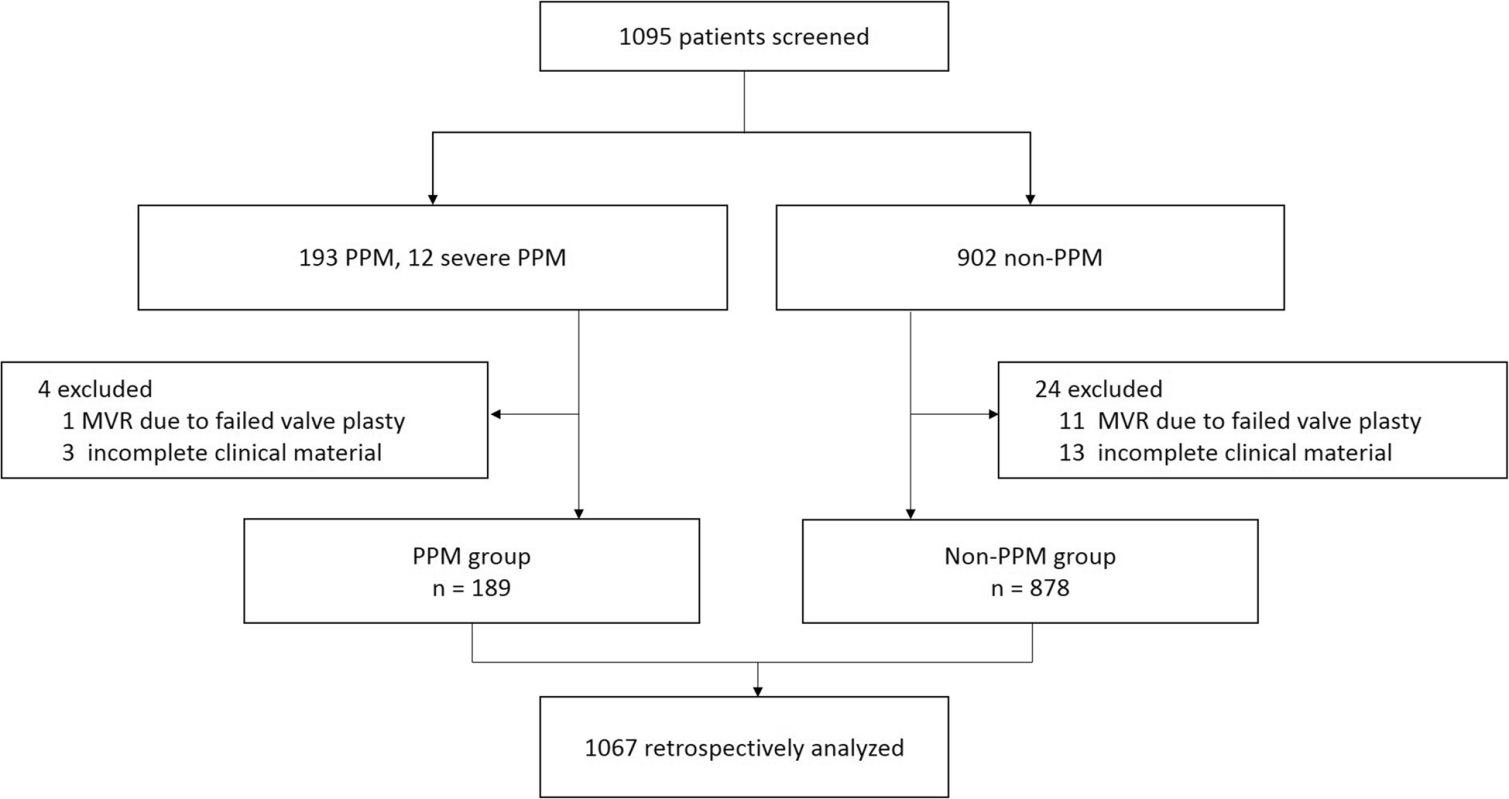
Consort flow diagram of patient enrollment

We analyzed all consecutive patients aged more than 18 years undergoing isolated MVR or MVR concomitant with other non-valve-replacement procedures. Patients with incomplete clinical data or patients who received MVR due to failed mitral valvuloplasty were excluded (Fig. 1).

In total, 1067 patients were included in this study. Baseline, intraoperative and outcome data were prospectively collected and validated, which were queried retrospectively. 30-month postoperative follow-up was conducted for discharged patients at the outpatient clinic. Patients who did not show up at the visit were contacted by telephone.

#### PPM definition and EOA index (EOAi) calculation

Body surface area (BSA) of the patients was calculated using the Dubois formula. The EOA of the mitral valve prosthesis was derived from in vitro measurements provided by the manufacturers and from scientific publications, as outlined in Table 1.

**Table 1:** In vivo effective orifice area values (cm^2^) corresponding to each valve

Valve prosthesis	patients	23 mm	25 mm	27 mm	29 mm	31 mm	33 mm	Ref
Mechanical	868							
CarboMedics Orbis Universal	680	1.8	2.2	2.2	2.4	2.4	2.3	[12]
St.Jude Master series	154	1.0	1.5	1.8	1.8	2.0	2.0	[13, 21]
ATS open pivot	33	–	1.8	2.8	2.8	2.9	2.9	[22]
Bioprosthesis	199							
Medtronics Hancock II	110	–	1.5	1.8	1.9	2.6	2.6	[12]
Medtronics Mosaic	5	–	1.5	1.7	1.9	1.9	–	[13]
St. Jude Bicor Stented	45	–	1.4	1.5	2.3	2.2	2.3	[23]
Carpentier-Edwards perimount	39	–	1.7	1.9	2.3	2.8	2.7	[12]

Ref: reference

EOAi (also called indexed effective orifice area, iEOA) was obtained with EOA divided by BSA. Mitral PPM was defined as EOAi ≤1.2 cm^2^/m^2^. EOAi ≤0.9 cm^2^/m^2^ was considered severe mitral PPM.

Other definitions were listed as follows. Chronic renal insufficiency: serum creatinine ≥2 mg/dl. Peripheral arterial disease: claudication, carotid stenosis > 50% or previous/planned intervention on the abdominal aorta, limb arteries or carotids. Coronary artery disease: ≥ 50% reduction in one or more coronary vessels in single or multiple plane angiographic images. Emergency surgery: operation required within 24 h of onset of symptoms. Postoperative renal failure: increase in baseline creatinine greater than 2 mg/dl.

### Surgical technique and prosthesis application

The operation records of all patients were reviewed. A total of 868 mechanical valve prostheses and 199 bioprostheses were implanted. The following prostheses were used in as followings:

Mechanical prosthesis: CarboMedics Orbis Universal (CarboMedics, Inc., Austin, TX, USA) (*n* = 679); St Jude Master (St Jude Medical, Inc., St Paul, MN, USA) (*n* = 154); ATS open pivot (ATS Medical, Inc., Minneapolis, MN, USA) (*n* = 33); Bioprosthesis: Hancock II Porcine Bioprosthesis (Medtronic, Inc., Minneapolis, MN, USA) (*n* = 110); Mosaic Porcine Bioprosthetic Valves (Medtronic, Inc., Minneapolis, MN, USA) (*n* = 5); Bicor Stented Bioprosthesis (St Jude Medical, Inc., St Paul, MN, USA) (*n* = 45); Carpentier-Edwards perimount (Baxter Healthcare Corp., Edwards Division, Santa Ana, CA, USA) (*n* = 39).

An isolated or concomitant MVR was performed in all patients. Concomitant procedures included tricuspid valvuloplasty, coronary artery bypass grafting, atrial septum defect repair and/or other procedures. Standard anesthesia and cardiopulmonary bypass methods were implemented. Most of the patients were approached through a full median sternotomy followed by an antegrade 4:1 cold blood cardioplegia for myocardial protection. Antegrade plus retrograde cardioplegia was applied for patients with coronary stenosis. Intermittent perfusion of cold blood cardioplegia was maintained at a frequency of once every 20 min.

After consulting with the patients preoperatively, the final decision of the type of prosthesis was made by the surgeons during operation, taking into consideration the preoperative information and intraoperative findings. When performing the MVR, sub-valvular structures were preserved as much as possible.

#### Statistical analysis

The Kolmogorov-Smirnov test was used to verify the distribution of all the quantitative variables. Gaussian distributed continuous variables were presented as mean ± standard deviation (SD), while non-Gaussian distributed variables were presented as medians (interquartile range). Categorical variables were expressed as an absolute number (percentage). Pearson’s χ2 test was used for descriptive, univariate statistics, such as the comparison of portions, while the Student’s unpaired *t*-test was used for normally distributed data comparisons. Otherwise, the Mann-Whitney *U* test was otherwise used for comparison of non-Gaussian distributed variables. Two-tailed *P*-values were derived from the calculated test statistics, and *P* ≤ 0.05 was considered statistically significant. Binary multivariate logistic regression analysis was performed to study the factors affecting PPM as well as mortality. IBM SPSS Statistics 20.0 software (IBM, Armonk, NY, USA) was used to analyze the data.

## Results

### Preoperative data and baseline information

A total of 1067 patients were included in this study. Mitral PPM was detected in 17.71% (189/1067) of the patients and only 12 (1.12%) patients met the criteria for severe PPM.

Compared with the non-PPM group, patients with PPM were older, taller and heavier and had a higher prevalence of male gender, hypertension, smoking history, coronary heart disease, and had a lower prevalence of mitral stenosis (Table 2).

**Table 2:** Preoperative patient baseline information

preoperative information	total (*n* = 1067)	PPM group (*n* = 189)	non-PPM group (*n* = 878)	*P* value
Age, y	56(48–62)	63(55–67)	54(46–61)	< 0.001
Male	379(35.5%)	89(47.1%)	290(33.0%)	< 0.001
Height, cm	160.72 ± 7.82	163.26 ± 7.87	160.17 ± 7.71	< 0.001
Weight, kg	57.72 ± 10.33	62.42 ± 10.23	56.70 ± 10.07	< 0.001
BMI, kg/m^2^	22.29 ± 3.67	23.34 ± 2.86	22.07 ± 3.79	< 0.001
BSA, m^2^	1.57 ± 0.16	1.64 ± 0.17	1.55 ± 0.16	< 0.001
Smoking history	123(11.5%)	32(16.9%)	91(10.4%)	0.016
Diabetes	102(9.6%)	22(11.6%)	80(9.2%)	NS
Hypertention	192(18.0%)	47(24.9%)	145(16.5%)	0.009
Cerebrovascular accident	34(3.2%)	6(3.2%)	28(3.2%)	NS
Coronary heart disease	22(2.1%)	9(4.8%)	13(1.5%)	0.009
NYHA functional class (≥ III)	400(37.5%)	77(40.7%)	323(36.8%)	NS
Atrial fibrillation	533(50.0%)	92(48.7%)	441(50.2%)	NS
Previous cardiac surgery	54(5.1%)	8(4.2%)	46(5.2%)	NS
Previous MI	1(0.1%)	0(0.0%)	1(0.1%)	NS
Mitral stenosis	786(76.7%)	128(67.7%)	658(74.9%)	0.036
MR (moderate to severe)	470(44.0%)	88(46.5%)	382(43.5%)	NS
LVEF	62.11 ± 8.48	62.23 ± 8.95	62.09 ± 8.38	NS
LVdD, mm	50(45–56)	51(46–58)	50(45–56)	NS
LAD, mm	51.36 ± 12.04	51.36 ± 11.93	51.36 ± 12.07	NS
Emergency surgery	2(0.2%)	0(0.0%)	0(0.0%)	NS
Aspirin within 5 days	29(2.7%)	9(4.8%)	20(2.3%)	NS
Clopidogrel within 5 days	19(1.8%)	6(3.2%)	13(1.5%)	NS

*BMI* body mass index, *BSA* body surface area, *NYHA* New York Heart Association, *MI* myocardial infarction, *MR* mitral valve regurgitation, *LVEF* left ventricular ejection fraction, *LVdD* left ventricular diastolic diameter, *LAD* left atrial diameter, *NS* not significant

### Patient characterize, PPM and valve prosthesis size

**Fig. 2:**
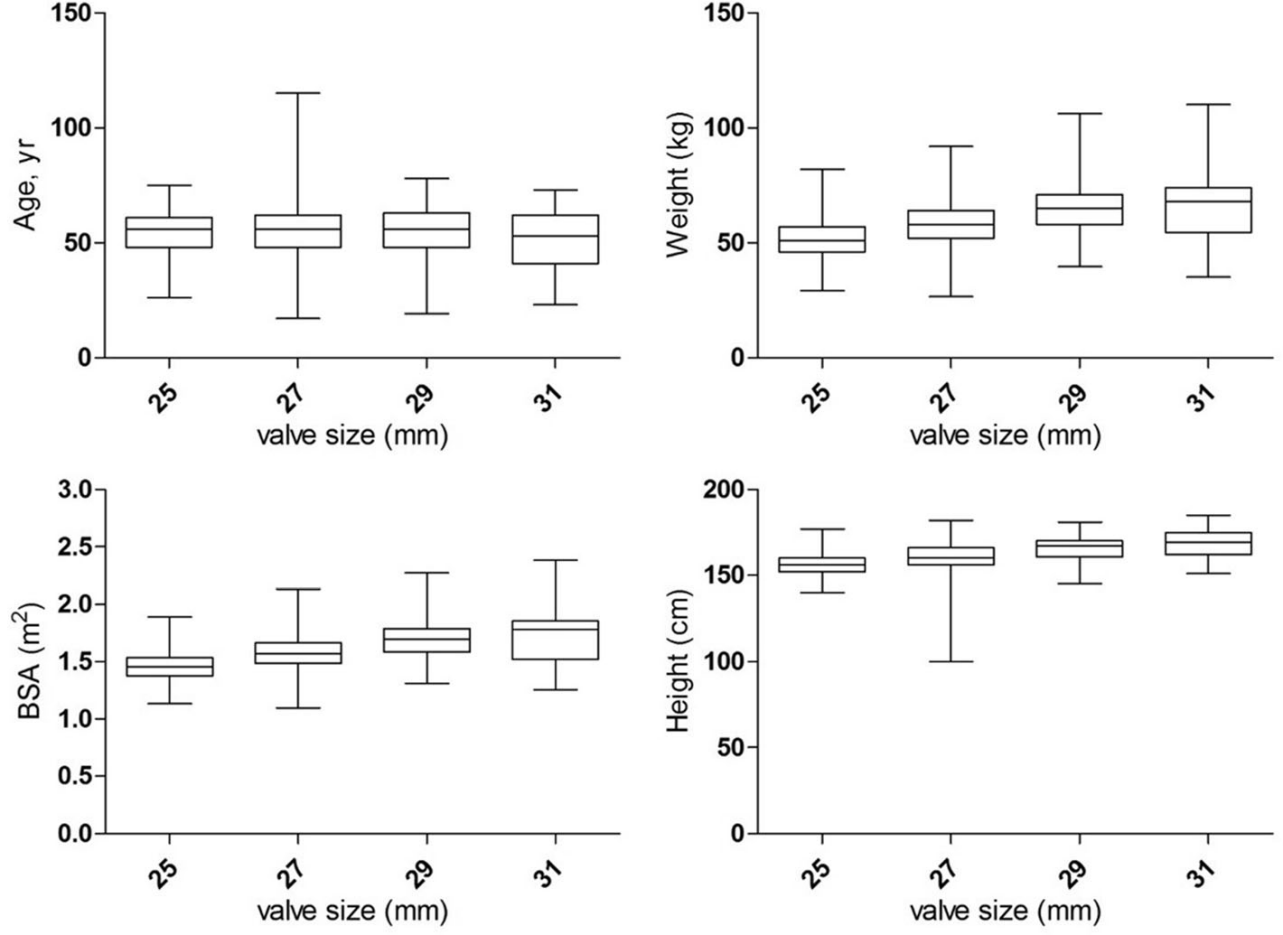
Boxplot showing distribution of age (NS), weight (*P* < 0.01), height (*P* < 0.01), and body surface area (*P* < 0.01), respectively, according to aortic valve size implanted

We analyzed the association among age, weight, height, BSA and valve size (Fig. 2). Krusal-Wallis analysis showed that weight, height and BSA are significantly associated with valve size (*P* < 0.01). In summary, larger mitral bioprosthetic valves were implanted in the taller and more obese patients.

**Fig. 3:**
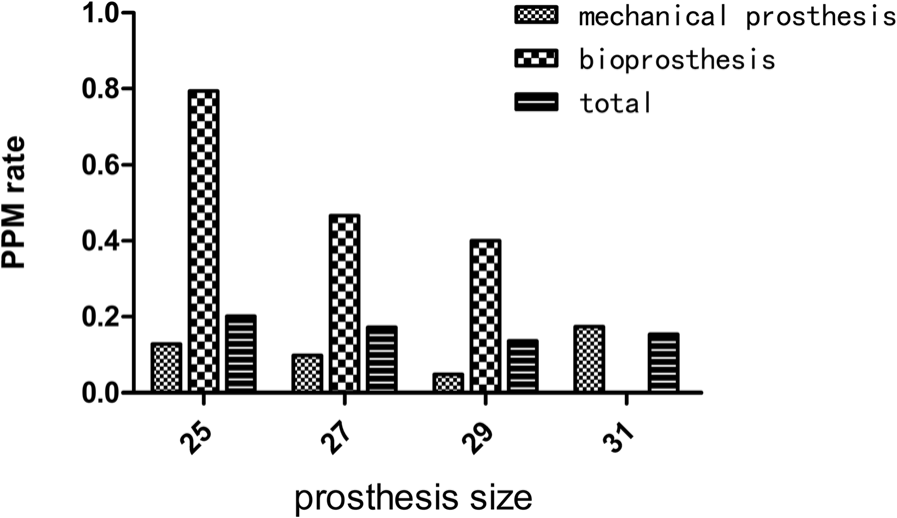
The PPM rate of each valve size for bioprostheses and mechanical prostheses respectively

Also, the PPM rate of each size of the prostheses was analyzed (Fig. 3). The results showed that the PPM rate of mechanical valve prostheses was considerably lower compared with bioprostheses (10.6% vs 48.5%, respectively, *P* < 0.001). As for the mechanical prostheses, there were no significant differences regarding the PPM occurrence of each valve size, whereas, the PPM rate of the 25 mm bioprosthesis was higher than that of the 27 mm and 29 mm bioprostheses (*P* < 0.01).

On the other hand, the PPM rate also differed among different brands of prostheses. As for mechanical prostheses, according to our data, PPM rate was highest (55.8%, 86/154) in patients underwent MVR with St. Jude Master Mechanical prostheses. And PPM rate was considerably low regarding CarboMedics mechanical prosthesis (0.9%, 6/679) and ATS open pivot mechanical prostheses (0.0%, 0/33). As for bioprosthesis, our results showed that PPM rate was high in patients using Medtronic Mosaic porcine bioprosthesis (80.0%, 4/5) or St. Jude Bicor bioprosthesis (82.2%, 37/45), whereas patients underwent MVR with Carpentier-Edwards Perimount bioprosthesis (17.7%, 5/38) and Medtronic Hancock II (45.4%, 50/110) showed lower rate of PPM.

### Operative data

As shown in Table 3, there were no significant differences between PPM and non-PPM patients regarding cardiopulmonary bypass (CPB) time. However, we found that there is an average five-minute cross-clamp time reduction in the PPM group.

**Table 3:** Intraoperative data

	total	PPM group *n* = 189	non-PPM group *n* = 878	*P* value
First time surgery	1013(94.9%)	181(95.8%)	832(94.7%)	NS
CPB time (min)	83(70–92)	83(70–89)	83(70–93)	NS
Cross-clamp time (min)	50(41–62)	45(40–55)	50(41–63)	< 0.001
Bioprosthesis	199(18.7%)	96(50.8%)	103(11.7%)	< 0.001
Combined procedure				
Tricuspid valve plasty	277(26.0%)	38(20.1%)	239(27.2%)	NS
CABG	28(2.6%)	11(5.8%)	17(1.9%)	0.005
AFRA or Maze surgery	106(9.9%)	30(15.9%)	76(8.7%)	0.005
Others	139(13.0%)	30(15.9%)	109(12.4%)	NS

*CPB* cardiopulmonary bypass, *CABG* coronary artery bypass grafting surgery, *AFRA* atrial fibrillation radio frequency surgery, *NS* not significant

Not surprisingly, remarkably more patients with a PPM were implanted with a bioprosthetic mitral valve. And as for patients who received a bioprosthesis, the prevalence of mitral stenosis was higher for mismatch patients (58.3% vs 41.7%, *P* = 0.019), whereas patients of mechanical prostheses did not differ in the prevalence of mitral stenosis, whether PPM or not (77.4% vs 79.4%, *P* > 0.05).

As for combined procedures, there were more combined coronary artery bypass grafting (CABG) and surgical ablation for atrial fibrillation in the PPM group.

### Factors affecting PPM

According to a multivariate binary logistic regression analysis including all preoperative and intraoperative variables, higher age (*P* = 0.011), larger BSA (*P* < 0.001), smaller left ventricular diastolic diameter (LVDd) (*P* < 0.001) and bioprosthesis (*P* < 0.001) were factors affecting mitral PPM (Table 4).

**Table 4:** Logistic regression analysis for prosthesis-patient mismatch

Factors	mean or %	OR	95% CI	*P* value
Age	54	1.029	1.006–1.051	0.011
BSA (m^2^)	1.57	152.111	45.261–511.208	< 0.001
LVDd (mm)	51.26	0.964	0.944–0.984	< 0.001
bioprosthesis	18.7%	7.539	4.632–12.273	< 0.001

*OR* odds ratio, *CI* confidence interval, *BSA* body surface area, *LVDd* left ventricular diastolic diameter

### Postoperative outcomes and factors affecting postoperative mortality

There were no obvious differences between the two groups regarding early post-operative complications including blood transfusion, ventilation time, reintubation, intensive care unit (ICU) time, postop stroke, postop atrial fibrillation and short-term mortality. Also, there were no other reoperation for valve-rated complications including PPM or other cardiac disease during hospital-stay except that two patients underwent emergency percutaneous coronary intervention for acute myocardium ischemia. Interestingly, we found that there was a small increase in hospitalization expense as well as a slightly prolonged hospital stay for the PPM patients (Table 5).

**Table 5:** Postoperative outcomes

	total	PPM group	non-PPM group	P
Perioperative transfusion	269(25.2%)	47(24.9%)	222(25.3%)	NS
Ventilation time (hr)	21(20–23)	21(20–23)	21(20–23)	NS
Reintubation	3(0.3%)	0(0.0%)	3(0.3%)	NS
Duration of first time ICU	72(72–96)	72(72–96)	72(72–96)	NS
Reentering ICU	2(0.2%)	2(1.1%)	0(0.0%)	NS
Chest tube output (ml)	545.88 ± 365.82	555.15 ± 295.51	543.87 ± 379.47	NS
Reoperation for bleeding	18(1.7%)	1(0.5%)	17(1.9%)	NS
Sternal wound infection	3(0.3%)	0(0.0%)	3(0.3%)	NS
Cerebral infarction	5(0.5%)	0(0.0%)	5(0.6%)	NS
Postoperative stroke	1(0.1%)	0(0.0%)	1(0.1%)	NS
Newly onset AF	3(0.3%)	0(0.0%)	3(0.3%)	NS
Mortality within 30 days	9(0.8%)	2(1.1%)	7(0.8%)	NS
Hospitalization expense (USD)	13,726 (11632–16,030)	14,446 (12538–17,010)	13,628 (11509–15,775)	0.032
Length of stay (d)	14(12–18)	15(13–19)	14(12–18)	0.011

*ICU* intensive care unit, *AF* atrial fibrillation, *NS* not significant

Altogether there were nine patients died within 30 days after surgery. Among them, five patients died due to malignant arrhythmia or cardiac arrest, two patient died of sever systematic infection, one patient died of uncontrollable bleeding and one patient died because of stroke. Among these short-term deaths, 2 patients underwent MVR with Hancock II bioprostheses, whereas 7 patients were replaced with CarboMedics mechanical prostheses.

Logistic regression analysis showed that smoking history and preoperative low left ventricular ejection fraction (LVEF) were independent factors predicting post-operative short-term all-cause mortality. However, PPM was not a risk factor for short-term mortality (Table 6).

**Table 6:** Logistic regression model for postoperative 30-day global mortaliy

Factors	mean or %	Odds ratio	95% CI	*P* value
smoking history	11.5%	3.199	0.729–14.041	0.004
preoperative LVEF	62.11	0.955	0.887–1.029	0.001
PPM	17.7%	1.138	0.226–5.743	0.654

*CI* confidence interval, *LVEF* left ventricular ejection fraction

### Mid-term follow-up

During mid-term follow-up, two patients underwent re-operative for stuck of the mechanical prostheses (both CarboMedics Mechanical prostheses). Both of the patients had an irregular medication history of Warfarin.

**Fig. 4:**
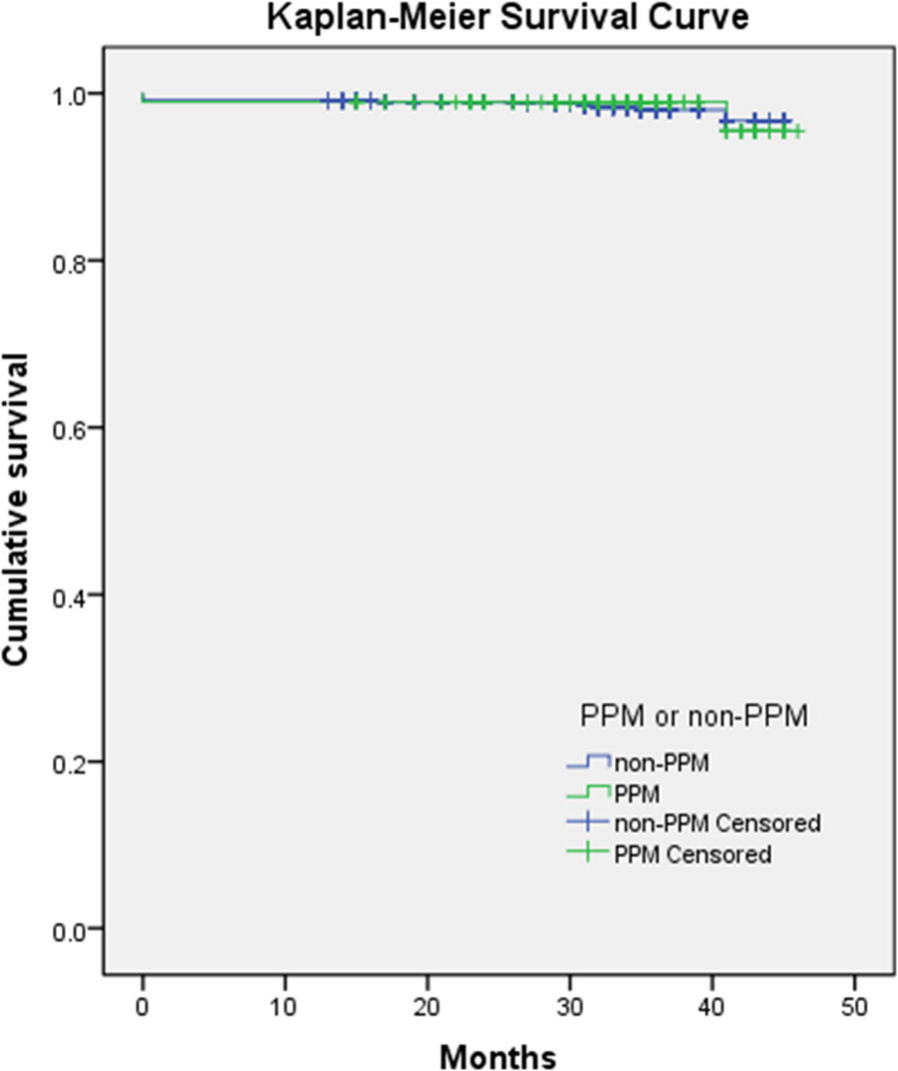
Kaplan-Meier cumulative mid-term survival, Prosthesis-patient mismatch (PPM) vs non prosthesis-patient mismatch

Mid-term deaths occurred in eight patients who all underwent MVR with a mechanical prosthesis, adding to the previously mentioned nine short-term deaths. Cumulative mid-term overall survival is 0.986 for both PPM and non-PPM patients (Fig. 4), and there were no significant difference regarding mid-term mortality for the two groups (SE 0.037, Log-rank *p* = 0.847). All the later occurring eight deaths were coagulation-related death. The overall mortality at 30 months was approximately 1.6% (Table 7). During follow-up, about 9.2% of the patients presented compromised cardiac functions with New York Heart Association (NYHA) functional classes III to IV. However, there were no significant differences between the PPM and non-PPM patients.

**Table 7:** Mid-term follow-up information

	Total	PPM group	non-PPM group	*P* value
Follow-up time (months)	31(23–35)	32(24–35)	31(23–33)	*P* = 0.362
NYHA functional class (III-IV)	9.2%	9.0%	9.6%	*P* = 0.902
Mortality	17(1.6%)	3(1.6%)	14(1.6%)	*P* = 0.994

*NYHA* New York Heart Association

## Discussion

### PPM occurrence and its risk factors

Although highly variable, PPM rates for mitral position in most of the literature ranged from 20 to 70% [11–16]. However, the incidence of PPM after MVR in our single-centered cohort was 17.7% and only 1.2% of the cases met the criteria for severe PPM.

We performed logistic regression analysis and found that larger BSA, higher age, implantation of bioprosthesis and smaller LVDd were risk factors for PPM. Besides BSA, EOA was the only variable defining the EOAi which determined the occurrence of PPM. Bioprosthesis possessed smaller EOA compared with mechanical prosthesis of the same valve size, leading to an increased rate of PPM. Also, bioprostheses were more prone to late degenerative calcification, which may further decrease its EOA. Thus, the more common use of bioprosthesis for degenerative mitral regurgitation might explain the lower prevalence of mitral stenosis of the PPM group in the preoperative data. As for LVDd, it was an indirect reflection of the mitral annulus diameter, which was another decisive factor in choosing prosthesis size, affecting the EOA of the prosthesis implanted. Higher age was then associated with more bioprosthesis implantation, thus leading to the increase in PPM occurrence. Hence, the difference of patient baseline characteristics between the PPM and non-PPM patients in hypertension and coronary heart disease could be explained by the higher age and obesity of the PPM patients.

In the Asian population, especially the eastern Chinese population, mitral stenosis and small size mitral prosthesis implantation might generally be considered to occur more frequently than in Western populations due to rheumatic causes associated with a small annulus. However, due to rheumatic etiology, the episode age of these patients was considerably younger than patients with valvular degeneration as predominant causes in Western countries. Thus, a larger ratio of patients of this population were implanted with mechanical mitral prostheses which possessed larger EOA than bioprosthesis. Also, patients of this population had a smaller body surface area than those in Western populations, leading to a further reduction in PPM occurrence. The aforementioned factors altogether help explain the low PPM rate in our study population.

### PPM and patient outcomes

Since its first description in 1978 by Rahimtoola [1], PPM after MVR has been suggested to potentially correlate with poor clinical outcomes including late tricuspid regurgitation and persistent pulmonary hypertension [11, 14, 17], similar to the outcomes of residual mitral stenosis. However, there were also reports suggesting that PPM did not affect survival after MVR [18, 19].

In our analysis, no impact of PPM on patient mortality was detected either in the postoperative short-term period or in the mid-term follow-up. Our findings are consistent with several large sample multi-centered analyses [15, 19]. Our results showed that smoking history and low preoperative LVEF were associated with higher short-term mortality, but not PPM.

Interestingly, our study showed that cross-clamp times were shorter in patients with PPM, with an average shortened time of 5 min. This might be explained because less time was spent suturing the mitral prosthesis due to the smaller mitral annulus diameter of the PPM patients. The longer hospitalization time of the PPM patients shown in the results might be due to the their higher average age and because their recovery time might be longer than in younger patients. Also, the elevated hospitalization expense could be explained by higher price of the bioprosthesis which was more common in the PPM group.

### Clinical implication for east Asian population

Currently, the most precise parameter in characterizing PPM is the EOAi [20], which is defined as the EOA of the prosthesis divided by the patient’s BSA. EOAi is in fact the only parameter found to consistently correlate with the postoperative gradient; therefore it is the most widely used. In Western countries, the predominant cause of mitral valve disease is degenerative mitral valve regurgitation. For this population, patients with mitral valve diseases usually have a larger left ventricle volume (left ventricular diastolic diameter) than the eastern Asian population; therefore, implantation of a large size prosthesis to avoid PPM will not have an obvious effect on left ventricular function. Hence, the parameter of EOAi has high feasibility in characterizing PPM for Western populations.

However, in a rheumatic population such as the eastern Asian population, the incidence of mitral valve stenosis is much higher than mitral valve regurgitation [11]. A larger proportion of this population has small left ventricle size, with part of the patients’ LVDd even smaller than the mitral annulus diameter. For these patients, implantation of a large sized prosthesis might compromise the effective cardiac muscular contraction of the left ventricle, causing left ventricular systolic dysfunction or, even worse, increase the risk of left ventricular rupture.

In our opinion, whether the current PPM standard is suitable for a rheumatic population such as the eastern Chinese is worth further exploration. Our results showed that there were no differences regarding the PPM occurrence of each valve size in the mechanical prosthesis. In our future study, we hope that we can explore a more precise parameter in predicting PPM then the current one, hence providing a more accurate prediction of patient outcomes for patients who underwent MVR in our population.

### Limitations of the study

There are limitations of the study which must be recognized. First, this is a retrospective analysis, and, as an inherent disadvantage, the recorded differences in patient outcomes could have originated from smaller recorded or unrecorded differences between PPM and non-PPM patients. Second, in our study, EOA was predicted by reference tables, which might not reflect the actual in vivo values of the EOAi. Moreover, this is a single-centered short/mid-term study, and sample size and follow-up time were limited. Therefore, a randomized prospective multi-centered clinical trial with a long follow-up time is needed to study the effect of mitral PPM on longer-term patient outcomes.

## Conclusions

Our results demonstrated that higher age, bioprosthesis, larger BSA and smaller left ventricle were associated with mitral PPM. However, PPM was not associated with poorer early outcomes after MVR surgery. In eastern China, the prevalence of mitral valve stenosis is high; therefore, whether the standard PPM criteria are suitable for patients of this district needs to be further verified.

## Abbreviations

AF: Atrial fibrillation
AFRA: Atrial fibrillation radio frequency surgery
BMI: Body mass index
BSA: Body surface are
CABG: Coronary artery bypass grafting
CABG: Coronary artery bypass grafting
CI: Confidence interval
CPB: Cardiopulmonary bypass
EOA: Effective orifice area
EOAi (iEOA): Effective orifice area index
ICU: Intensive care unit
LAD: Left atrial diameter
LVDd: Left ventricular diastolic diameter
LVEF: Left ventricular ejection fracture
LVESV: Left ventricular end-systolic volume
MR: Mitral valve regurgitation
MVR: mitral valve replacement
NS: Not significant
NYHA: New York Heart Association
OR: Odd ratio
PHM: Prosthesis-patient mismatch
PPM: Prosthesis-patient mismatch
SD: Standard deviation
